# Medicaid Coverage of Guidelines-Based Asthma Care Across 50 States, the District of Columbia, and Puerto Rico, 2016-2017

**DOI:** 10.5888/pcd15.180116

**Published:** 2018-09-06

**Authors:** Katherine Pruitt, Annie Yu, Barbara M. Kaplan, Joy Hsu, Pamela Collins

**Affiliations:** 1 American Lung Association, Washington, District of Columbia; 2 Centers for Disease Control and Prevention, National Center of Environmental Health, Atlanta, Georgia

## Abstract

Asthma affects more than 24 million Americans, including 6.2 million children. Although asthma cannot be cured, it can be effectively managed with care based on nationally recognized guidelines. Ensuring the availability and accessibility of guidelines-recommended treatments and services can help patients receive the most appropriate care. In this article, we describe the American Lung Association’s Asthma Guidelines-Based Care Coverage Project (the Project) to determine the extent of asthma care coverage and associated barriers in state Medicaid programs — information that has been previously unavailable. The Project tracked coverage for 7 areas of guidelines-based asthma care and 9 barriers related to accessing care in Medicaid programs for all 50 states, the District of Columbia, and Puerto Rico. Results from the Project show a lack of consistent and comprehensive coverage across states, as well as coverage-related challenges to accessing asthma care within states.

## The Burden of Asthma in the United States

Treatments and services are necessary to control and manage asthma, which affects more than 24 million Americans, including 6.2 million children (1). Without proper treatment, asthma can be dangerous, even fatal. Poorly managed asthma resulted in 1.6 million emergency department visits in 2013 (2) and 439,000 hospitalizations in 2010 (3). Asthma accounts for $82 billion in national health care costs annually (4).

Medicaid is a substantial source of coverage for people living with asthma: children with asthma are more likely to have coverage through Medicaid and the Children’s Health Insurance Plan than children without asthma (47.4% vs 38.1%) (5), and adults aged 18 to 64 years in Medicaid have asthma at a rate almost twice as those with private insurance (13.1% vs 7.1%) (5). The objective of this analysis is to describe the extent of asthma care coverage and associated barriers in state Medicaid programs in all 50 states, the District of Columbia (DC), and Puerto Rico.

## The Asthma Guidelines-Based Care Coverage Project

The Asthma Guidelines-Based Care Coverage Project (the Project) arose from the recognition that adhering to guidelines-based treatment can reduce the burden of asthma and improve patient outcomes (6). Best practices for asthma care, in the context of this project, were based on recommendations in the National Asthma Education and Prevention Program (NAEPP) Expert Panel Report 3 (7). Before the Project, to what extent guidelines-based care was covered by state Medicaid programs was unknown. To answer that question, the American Lung Association conducted a comprehensive analysis of Medicaid coverage of 7 categories of care and 9 barriers to accessing care in all 50 states, the District of Columbia, and Puerto Rico.

Project staff collected data on 7 categories of care: quick relief medication (4 types), controller medication (20 types), medical devices (3 types), allergy testing (2 types), allergen immunotherapy (also known as “allergy shots”), home visits, and asthma self-management education. Project staff also tracked 9 barriers to accessing care: age limits, age restrictions, copayment, durable medical equipment benefits, prior authorization, quantity limits, step therapy, specialty visit limitations, and eligibility criteria (Table 1)*. *Comprehensive and consistent guidelines-based asthma care was defined as coverage of all categories of care without any barriers.

**Table 1:** Categories of Care and Barriers to Accessing Asthma Care Defined, American Lung Association’s Asthma Guidelines-Based Care Coverage Project, 50 US States, the District of Columbia, and Puerto Rico

Terminology	Definition
**Categories of Care**	
Quick relief medications	Fast acting or quick relief medications intended to provide immediate relief of bronchoconstriction and its accompanying acute symptoms.
Controller medications	Long-term control medications taken daily on a long-term basis to control persistent asthma.
Devices	Devices that assist with monitoring symptoms or properly administering asthma medication (includes nebulizers, peak flow meters, and valved-holding chambers).
Allergen testing	Allergy tests for asthma triggers to identify and reduce exposure to allergens (skin or in vitro testing).
Allergen immunotherapy	Preventive treatment through incremental injected or ingested increases of the allergen to make the immune system less sensitive to the substance.
Home visits	Education, assessment, and intervention of the patient’s home environment to address triggers.
Asthma self-management education	Patients and families learn to use prescribed asthma-control medicines and equipment correctly, recognize symptoms of an asthma episode and respond appropriately and mitigate asthma triggers.
**Barriers**	
Age limits	This barrier indicates that the treatment is only covered if a patient is under the age of x and applied only if it is more restrictive than FDA-approved guidelines.
Age restrictions	This barrier indicates that the treatment is only covered for patients over the age of x and applied only if it is more restrictive than FDA-approved guidelines.
Copayments	Payment that must be made to receive the treatment even when it is covered by the health plan (in this case Medicaid or Medicaid-managed care plans).
DME	A device is covered only as DME, which could result in having to pay full price for the device at a retail pharmacy.
Eligibility criteria	A plan will only provide the treatment after a patient has experienced an incident, such as numerous visits to the emergency department.
Prior authorization	This barrier requires the provider to get approval from the insurance company (in this case Medicaid or Medicaid-managed care plans) before the treatment will be covered (ie, paid for).
Quantity limits	There is a limit on the number of treatments covered each month or under a certain amount of time.
Specialty visit limitations	This is when a plan only allows a patient to see a fixed number of specialists per year.
Stepped therapy	This means a plan requires a patient to try and fail on a different treatment before the insurance company (in this case Medicaid or Medicaid-managed care plans) will pay for the treatment that his/her provider prescribes.

Abbreviations: DME, durable medical equipment; FDA, Food and Drug Administration.

To collect data on state Medicaid program coverage of and barriers to comprehensive asthma guidelines-based care, the American Lung Association (Lung Association) examined publicly available documents such as Medicaid state plans (including managed care plans), formularies, preferred drug lists, member handbooks, and other related information for every state Medicaid program. Project staff then confirmed data findings with each state Medicaid office to ensure accuracy. (Complete state-by-state findings are published on the American Lung Association website at www.lung.org/asthma-care-coverage.) The findings discussed in this article reflect data collected as of June 30, 2017.

The Lung Association analyzed asthma treatments and services covered by each state Medicaid plan and compiled them to determine the state’s overall Medicaid program coverage for the category. Coverage values were generated to represent coverage across all Medicaid plans within the state (Table 2). If the state Medicaid program covered the treatment or service in any capacity, barrier data were also assessed. Possible barrier occurrences varied for each treatment or service depending on how many state Medicaid programs provided coverage. Barriers for each treatment and service were grouped into their respective categories of care to capture how common the barriers were for the category. The frequency of applicable barriers was calculated for each category on the basis of how often the barrier appeared (either in every plan in the state or some plans for that category) and how much barrier data were available (total possible barrier occurrences based on coverage). Frequency allowed for comparability of barriers across the categories of care to determine the most common barriers (Table 3).

**Table 2:** State Medicaid Program Coverage of Guidelines-Based Asthma Care Categories, American Lung Association’s Asthma Guidelines-Based Care Coverage Project, 50 US States, the District of Columbia, and Puerto Rico^a^

State	Quick Relief Medication (4 Types)	Controller Medication (20 Types)	Medical Devices (3 Types)	Allergy Testing (2 Types)	Allergen Immunotherapy	Home Visits	Asthma Self-Management Education
Alabama	Y	Y	V	Y	Y	Y	Y
Alaska	Y	V	N	N	N	N	Y
Arizona	V	V	V	V	V	N	V
Arkansas	Y	V	N	N	N	N	N
California	V	V	V	V	V	N	V
Colorado	V	V	Y	V	V	N	N
Connecticut	Y	Y	Y	Y	Y	Y	Y
Delaware	Y	V	Y	Y	Y	V	Y
Florida	V	V	V	V	V	V	V
Georgia	V	V	Y	Y	Y	V	V
Hawaii	V	V	V	V	V	N	V
Idaho	Y	Y	Y	Y	Y	N	Y
Illinois	V	V	V	V	V	N	V
Indiana	Y	V	V	V	V	V	V
Iowa	Y	V	Y	Y	Y	N	N
Kansas	Y	Y	V	Y	Y	Y	Y
Kentucky	Y	V	V	Y	Y	N	V
Louisiana	V	V	V	V	V	V	V
Maine	Y	Y	Y	Y	Y	N	Y
Maryland	V	V	V	V	V	V	V
Massachusetts	V	V	V	V	V	V	V
Michigan	V	V	V	V	V	V	V
Minnesota	V	V	V	V	Y	V	V
Mississippi	Y	V	V	V	N	N	N
Missouri	Y	V	NA	V	V	N	V
Montana	Y	V	Y	Y	Y	N	Y
Nebraska	Y	V	V	Y	Y	N	V
Nevada	V	V	V	V	V	N	Y
New Hampshire	V	V	V	Y	Y	N	NA
New Jersey	V	V	V	N	N	N	N
New Mexico	V	V	V	N	V	N	N
New York	V	V	V	V	V	N	V
North Carolina	Y	Y	V	Y	Y	N	Y
North Dakota	Y	Y	Y	V	Y	N	N
Ohio	V	V	V	V	V	N	N
Oklahoma	Y	Y	V	Y	Y	N	N
Oregon	V	V	V	V	V	V	V
Pennsylvania	V	V	V	V	V	V	V
Rhode Island	V	V	V	Y	Y	N	Y
South Carolina	V	V	V	V	V	V	V
South Dakota	N	V	Y	N	N	N	N
Tennessee	Y	V	V	N	V	N	N
Texas	Y	V	V	V	V	V	V
Utah	V	V	V	V	V	N	V
Vermont	Y	V	Y	Y	Y	N	Y
Virginia	V	V	V	V	V	V	N
Washington	V	V	V	Y	Y	V	V
West Virginia	Y	V	V	V	N	N	N
Wisconsin	V	V	V	N	N	N	N
Wyoming	Y	Y	Y	Y	Y	Y	N
District of Columbia	V	V	V	V	V	N	V
Puerto Rico	V	V	V	Y	Y	V	Y
**Totals**							
**Yes**	23	9	12	19	21	4	13
**Varies**	28	43	37	26	24	16	23
**No**	1	0	2	7	7	32	15
**NA**	0	0	1	0	0	0	1

a N indicates that none of the items in the category is covered in any Medicaid plan; NA indicates that data were not available for the state; V indicates that not all items in the category were covered in all Medicaid plans (some items in category may have varying coverage across plans or no coverage at all); and Y indicates that all items in the category of care are covered in all Medicaid plans. Data collected as of June 30, 2017.

**Table 3:** Frequency^a^ and Total Possible Occurrences of Barriers for Treatments and Services Among State Medicaid Programs Covering the Category of Care, American Lung Association’s Asthma Guidelines-Based Care Coverage Project, 50 US States, the District of Columbia, and Puerto Rico^b^

Barriers	Quick Relief Medication	Controller Medication	Medical Devices	Allergy Testing	Allergen Immunotherapy	Home Visits	Asthma Self-Management Education
	% (No.)						
Age limits	4.0 (149)	10.2 (749)	13.0 (69)	0 (29)	0 (15)	0 (8)	0 (10)
Age requirement	1.4 (147)	4.5 (734)	0 (64)	0 (29)	6.3 (16)	0 (8)	0 (10)
Copayment	74.7 (178)	73.1 (886)	58.2 (67)	53.7 (41)	52.6 (19)	0 (8)	20.0 (10)
Durable medical equipment benefits	NA	NA	71.3 (94)	NA	NA	NA	NA
Eligibility criteria	0.7 (148)	1.9 (734)	5.8 (69)	0 (29)	0 (15)	0 (8)	0 (10)
Prior authorization	26.4 (163)	39.2 (824)	32.5 (80)	28.1 (32)	23.5 (17)	22.2 (9)	9.1 (11)
Quantity limits	58.7 (172)	52.2 (820)	68.7 (99)	28.6 (35)	31.3 (16)	22.2 (9)	9.1 (11)
Specialty visit limitation	NA	NA	NA	0 (14)	0 (15)	0 (8)	0 (10)
Step therapy	17.7 (153)	23.6 (768)	NA	0 (29)	0 (15)	0 (8)	0 (10)

a Frequency of a barrier is the occurrence of the barrier divided by the total possible occurrences of the barrier.

b Data collected as of June 30, 2017.

## Current Landscape: Coverage and Barriers

### Coverage

As shown in Table 2, most state Medicaid programs provide some form of coverage for the 7 categories of guidelines-based asthma care (Table 2). Quick relief medication was the most widely covered treatment: 23 state Medicaid programs covered all types of quick relief medication, 28 state Medicaid programs had varied coverage by plan, and only one state Medicaid program did not cover quick relief medication. Home visits was the least covered treatment: only 4 state Medicaid programs provided coverage in all plans, 16 state Medicaid programs had varied coverage, and 32 state Medicaid programs did not cover home visits.

The most frequent coverage value found was “varies by plan.” Across all 7 categories of care, state Medicaid programs were more likely to have varied and inconsistent coverage than either full coverage or no coverage at all. State coverage that varied by plan was prevalent for controller medication (43 states) and medical devices (37 states).

**Figure 1:**
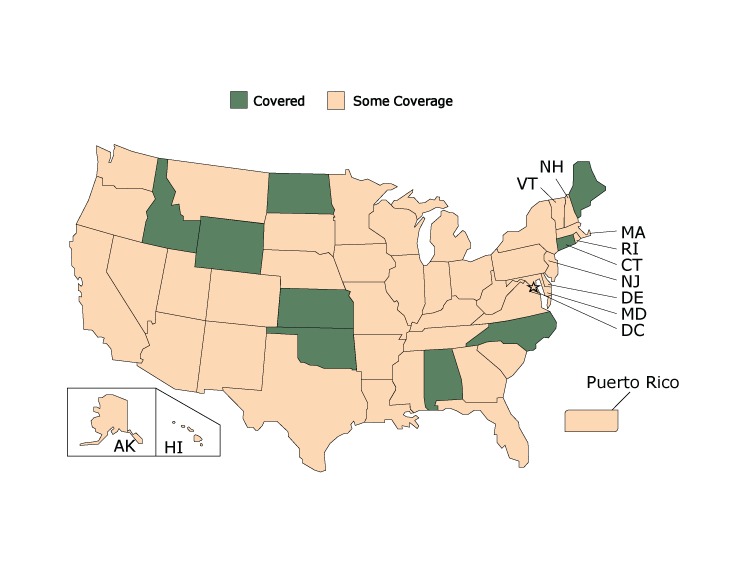
State Medicaid Coverage for Controller Medications, American Lung Association’s Asthma Guidelines-Based Care Coverage Project, 50 US States, the District of Columbia, and Puerto Rico. Data collected as of June 30, 2017. All states were either covered or had some coverage; no states were covered without barriers or had no coverage. **Table:** 

State Medicaid coverage was inconsistent for controller medications, both between and within states (Figure 1). Only 9 state Medicaid programs covered all controller medications.

State	Controller Medications Coverage
Alabama	Covered
Alaska	Some coverage
Arizona	Some coverage
Arkansas	Some coverage
California	Some coverage
Colorado	Some coverage
Connecticut	Covered
Delaware	Some coverage
Florida	Some coverage
Georgia	Some coverage
Hawaii	Some coverage
Idaho	Covered
Illinois	Some coverage
Indiana	Some coverage
Iowa	Some coverage
Kansas	Covered
Kentucky	Some coverage
Louisiana	Some coverage
Maine	Covered
Maryland	Some coverage
Massachusetts	Some coverage
Michigan	Some coverage
Minnesota	Some coverage
Mississippi	Some coverage
Missouri	Some coverage
Montana	Some coverage
Nebraska	Some coverage
Nevada	Some coverage
New Hampshire	Some coverage
New Jersey	Some coverage
New Mexico	Some coverage
New York	Some coverage
North Carolina	Covered
North Dakota	Covered
Ohio	Some coverage
Oklahoma	Covered
Oregon	Some coverage
Pennsylvania	Some coverage
Rhode Island	Some coverage
South Carolina	Some coverage
South Dakota	Some coverage
Tennessee	Some coverage
Texas	Some coverage
Utah	Some coverage
Vermont	Some coverage
Virginia	Some coverage
Washington	Some coverage
West Virginia	Some coverage
Wisconsin	Some coverage
Wyoming	Covered
District of Columbia	Some coverage
Puerto Rico	Some coverage

No state Medicaid program had plans that consistently covered all 7 categories of care without barriers. Connecticut covered all 7 categories of care but had barriers for some categories. Five state Medicaid programs (Alabama, Idaho, Kansas, Maine, and Wyoming) covered 6 of the 7 categories of care across all plans, the most of any state, albeit with barriers. Most states had a mix of coverage, with some programs covering several categories but having coverage that varied in other categories or having no coverage in some categories. All states had some coverage for at least one of the medication categories.

### Barriers

**Figure 2:**
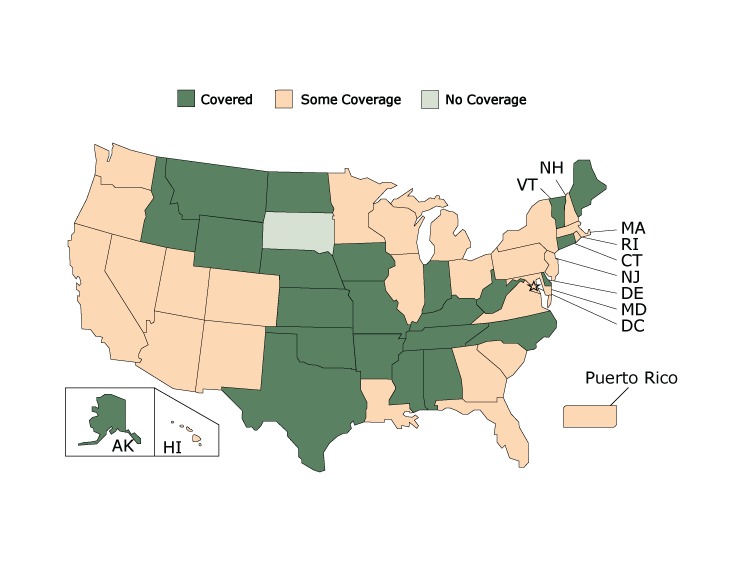
State Medicaid Coverage for Quick Relief Medications, American Lung Association’s Asthma Guidelines-Based Care Coverage Project, 50 US States, the District of Columbia, and Puerto Rico. Data collected as of June 30, 2017. All states were covered, had some coverage, or were not covered; no states were covered without barriers. **Table:** 

Even if a state covers all treatments in a care category, it is still very likely to have barriers to accessing the treatments. Figure 2 displays coverage for quick relief medications. Nearly half (23 state Medicaid programs) covered all quick relief medication treatments. However, none of these states covered all quick relief medications without any barriers.

State	Quick Relief Medications Coverage
Alabama	Covered
Alaska	Covered
Arizona	Some coverage
Arkansas	Covered
California	Some coverage
Colorado	Some coverage
Connecticut	Covered
Delaware	Covered
Florida	Some coverage
Georgia	Some coverage
Hawaii	Some coverage
Idaho	Covered
Illinois	Some coverage
Indiana	Covered
Iowa	Covered
Kansas	Covered
Kentucky	Covered
Louisiana	Some coverage
Maine	Covered
Maryland	Some coverage
Massachusetts	Some coverage
Michigan	Some coverage
Minnesota	Some coverage
Mississippi	Covered
Missouri	Covered
Montana	Covered
Nebraska	Covered
Nevada	Some coverage
New Hampshire	Some coverage
New Jersey	Some coverage
New Mexico	Some coverage
New York	Some coverage
North Carolina	Covered
North Dakota	Covered
Ohio	Some coverage
Oklahoma	Covered
Oregon	Some coverage
Pennsylvania	Some coverage
Rhode Island	Some coverage
South Carolina	Some coverage
South Dakota	No coverage
Tennessee	Covered
Texas	Covered
Utah	Some coverage
Vermont	Covered
Virginia	Some coverage
Washington	Some coverage
West Virginia	Covered
Wisconsin	Some coverage
Wyoming	Covered
District of Columbia	Some coverage
Puerto Rico	Some coverage

Barriers existed across states in all 7 categories of care (Table 3). Only 4 categories of care had any state Medicaid programs that covered the treatment without any barriers, and even then coverage without barriers was a minority: allergen immunotherapy (6 states), allergy testing (6 states), home visits (3 states) and asthma self-management education (6 states).

Copayment was the most common barrier assessed across all categories, especially for quick relief medications and controller medications. Copayment was observed in 74.7% of state Medicaid programs covering quick relief medications (133 of 178 possible occurrences of the barrier) and 73.1% of state Medicaid programs covering controller medications (648 of 886 possible occurrences of the barrier). Copayment was the most common barrier observed in 6 of the 7 categories of care except for home visits.

Quantity limit restrictions and prior authorization requirements were also common barriers observed in state Medicaid programs. Similar to copayment, quantity limit restrictions were mainly observed for coverage of quick relief medications (58.7%), controller medications (52.2%), and medical devices (68.7%). Prior authorization requirements were also most commonly observed for coverage of quick relief medications (26.4%), controller medications (39.2%), and medical devices (32.5%). With the exception of durable medical equipment benefits, which was observed in 71.3% of state Medicaid programs covering medical devices, copayment, quantity limits, and prior authorization were observed much more often than the other 5 barriers, where frequency of the barriers ranged from 0% to 23.6%.

## Opportunities to Improve Guidelines-Based Asthma Care Coverage and Access

Improving access to guidelines-based asthma care can result in better patient outcomes, including reduced asthma exacerbations and associated health care costs. However, the findings of this project show that there are substantial gaps between guidelines-based asthma care and coverage by state Medicaid programs. Many programs do not cover the recommended categories of care and have inconsistent coverage across fee-for-service and managed care plans within the same state, making it difficult for providers and patients to understand what asthma treatments and services are covered. Furthermore, the Project found that although some states may cover all treatments in a category, very few states had complete coverage of that category without barriers.

Some states show varying coverage in part because of the large number of managed care organizations in the state that contract with Medicaid. If one managed care plan did not cover all the medications in the treatment category but all others did, the state would still show varying coverage. Variable and inconsistent coverage across plans can impede access to asthma care treatments and services. A lack of consistent coverage may cause confusion and a lack of awareness among both patients and providers on what asthma care is available for the patient. Providers may not prescribe a treatment even if it is covered and reimbursed, because they do not know if the treatment is covered under the patient’s health plan, especially in states with many managed care plans that differ in coverage. What gets paid for gets done, but providers may not know if the asthma treatment or service is billable to Medicaid. Similarly, patients may not know what treatments are available to them under their health plan and may not seek care that could be more effective for their condition. The lack of consistent coverage impedes the promotion of covered treatments to Medicaid enrollees and use of guidelines-based treatments.

Barriers are a challenge to guidelines-based asthma care in the United States. Quick relief medications, controller medications, and medical devices had the greatest proportion of observed barriers. This finding may be because of the number of medications and devices that fall under these categories and because of the 3 categories being the most widely covered treatment methods of the 7 categories of care among state Medicaid programs.

The most common barrier observed in the Project was copayment, especially for quick relief medications (74.7%) and controller medications (73.1%), because of the large number of medications that fall under these 2 categories (4 quick relief medications and 20 controller medications) and the frequency with which these categories are used to treat asthma compared with the other categories. Copayment was observed for 6 of the 7 categories of care except for home visits, likely because of the small number of Medicaid programs that cover home visits and the unavailability of barrier information for the Medicaid programs that do cover the service. Cost-sharing, such as copayments, can reduce prescription drug use (8). Publicly insured populations (eg, those that rely on Medicaid) can be susceptible to medication nonadherence when required to pay for medications (9). Reduced use of guidelines-recommended asthma medication by asthma patients negatively affects their health outcomes.

Asthma control can be improved through adherence to guidelines-recommended pharmacotherapy and active engagement with self-management behaviors (10). Although many states show some coverage of these asthma care components, coverage often varies by plan and only a few states have coverage without any barriers. Barriers are associated with reduced medication adherence, such as discontinuation and diminished persistency (11). These barriers can make it difficult for patients to access the asthma care treatments they need in a timely manner, contributing to increased symptoms, health care use, and rates of morbidity and mortality. Removing these barriers can make it easier for patients to obtain medications and receive appropriate treatment.

Opportunities exist for both state Medicaid offices and Medicaid plans to remedy the disparity between the NAEPP guidelines (7) and what is covered by these plans. The Project’s data can help state Medicaid programs identify gaps in their asthma care coverage and improve coverage and access for Medicaid patients with asthma. State Medicaid programs can consider increasing the adoption of guidelines-based asthma care by ensuring consistent coverage of all treatments across plans and removing barriers impeding access to asthma care. State Medicaid programs can also consider assessing the extent to which providers are aware of and use covered services; increasing provider knowledge of covered services can increase use and provide better asthma care for patients. Consistent coverage of all treatments across plans and removal of barriers can reduce uncertainty about coverage by providers and help providers better connect patients with appropriate treatment and services. Medicaid programs that take these actions can substantially improve asthma patient outcomes and reduce costly exacerbations among Medicaid enrollees. 

The findings in this report are subject to limitations. Initial data collection was limited to publicly available documents to determine medical benefits. Some documentation of coverage may not be publicly accessible. Second, state Medicaid agency staff were contacted to review and confirm findings. Project staff did not directly contact the managed care organizations within states to review and confirm the findings for the managed care plans. Lastly, Medicaid staff in 19 states were unable to review and confirm data. In these states, findings were presented on an as-is basis based on publicly available documents. Thus, the extent of some states’ coverage is unknown.

The Project continues to assess coverage of asthma care treatments and services to track changes over time. By increasing available information about coverage in each state and increasing awareness of gaps in coverage, the Project aims to educate states about their Medicaid programs’ coverage of asthma care and encourage state Medicaid programs to cover all 7 categories of asthma care without any barriers so that asthma patients can receive the care they need.
